# Association Between Spikes in External Training Load and Shoulder Injuries in Competitive Adolescent Tennis Players: The SMASH Cohort Study

**DOI:** 10.1177/19417381211051643

**Published:** 2021-10-25

**Authors:** Fredrik Johansson, Ann Cools, Tim Gabbett, Jaime Fernandez-Fernandez, Eva Skillgate

**Affiliations:** † Tennis Research and Performance Group, MUSIC, Department of Health Promotion Sciences, Sophiahemmet University, Stockholm, Sweden; ‡ Unit of Intervention and Implementation Research for Worker Health, Institute of Environmental Medicine, Karolinska Institutet, Stockholm, Sweden; § Naprapathögskolan–Scandinavian College of Naprapathic Manual Medicine, Stockholm, Sweden; ‖ Department of Rehabilitation Sciences, Faculty of Medicine and Health Sciences, Ghent University Hospital, Ghent, Belgium; ¶ Gabbett Performance Solutions, Brisbane, Clayfield, Queensland, Australia; # Centre for Health Research, University of Southern Queensland, Ipswich, Queensland, Australia; ** Department of Physical Activity and Sports Sciences, Universidad de León, León, Spain

**Keywords:** workload, acute:chronic workload ratio (ACWR), tennis, adolescent, shoulder, injury

## Abstract

**Background:**

Few studies have examined the association between the acute:chronic workload ratio (ACWR) and complaints/injuries in young tennis players. Primary aims of this study were to investigate if accumulated external workload “spikes” in ACWR of tennis training, match play, and fitness training, and to see if high or low workload/age ratio were associated with the rate of shoulder complaints/injuries in competitive adolescent tennis players. Additional aims were to report the incidence of complaints/injuries stratified by sex and level of play and to describe shoulder injury characteristics.

**Hypothesis:**

Rapid increases in external workload are associated with the incidence of shoulder complaints and injuries.

**Study Design:**

A cohort study.

**Level of Evidence:**

Level 3.

**Methods:**

At baseline, 301 adolescent competitive tennis players, 13 to 19 years, were screened and followed weekly for 52 weeks with questionnaires, in the years 2018 to 2019. Information about time-varying accumulated external workload spikes (uncoupled ACWR >1.3), and workload/age ratio, in 252 uninjured players were used in Cox regression analyses with the outcomes shoulder complaints (≥20) and injuries (≥40) (Oslo Sports Trauma Research Center Overuse Injury Questionnaire).

**Results:**

For each additional workload spike in tennis training/match play, the hazard rate ratio (HRR) was 1.26 (95% CI, 1.13-1.40) for a shoulder complaint and 1.26 (95% CI, 1.15-1.39) for a shoulder injury. The HRR for fitness training was 1.11 (95% CI, 1.02-1.20) for a shoulder complaint and 1.18 (95% CI, 1.09-1.27) for a shoulder injury. Workload/age ratio was not associated with the rate of shoulder complaints or injuries.

**Conclusion:**

Accumulated external workload spikes of tennis training, match play, and/or fitness training are associated with a higher rate of shoulder complaints and shoulder injuries in competitive adolescent tennis players.

**Clinical Relevance:**

Consistency in training load on a weekly basis is most likely more beneficial for adolescent tennis players regarding shoulder complaints/injuries than a training schedule comprising rapid increases (ie, spikes) in workload.

One of the greatest challenges across all sports and for all athletes at the elite level is to optimize workload, minimize injury risk, and enhance performance.^21,39^ Adolescent athletes may spend more hours per week in sports than years they are old and may therefore be at risk of any injury.^24^ In this regard, sport specialization and intensive training during growth stages represent potential risk factors for overuse injuries in young athletes, which may reduce long-term performance and hinder the development of a professional career.^12^

In overhead sports, shoulder injuries and shoulder pain pose substantial problems for athletes, and several studies have presented a variety of risk factors such as decreased shoulder strength, range of motion deficits, and scapular dyskinesia that increase the risk of these complaints.^6,28,31,38,40,43,44^ Based on previous research (ie, cross-sectional data, prospective studies, and systematic reviews), shoulder injuries have been identified as one of the most common in young tennis players,^1,2,13,15,19,25,29,37,41^ with an incidence of 2.6 to 3.6 injuries per 1000 hours played.^17^

Over the past decade, the acute:chronic workload ratio (ACWR) and its association with noncontact and contact injuries has been extensively investigated. However, most studies are performed in team sports such as cricket, baseball, rugby, soccer, and handball.^4,11,20,21,31^ Apart from on-court tennis practice and fitness, tournament scheduling and participation in multiple draws often require young tennis players to complete numerous training sessions and/or competitive matches on consecutive days,^34^ or even 2 consecutive tennis matches or more in a day.^16^ Thus, the need for adequate training/competition monitoring as well as the implementation of recovery and rest are paramount for these young tennis athletes.

External training load describes any external training stimulus applied to an athlete (ie, distance covered, duration and frequency of training/competition) with internal training load comprising the psychophysiological response (ie, heart rate, rating of perceived exertion, blood lactate) to the external load.^22^ Research has shown that the ACWR using either internal training load and/or external training load is significantly associated with injury risk.^3^ In addition, considering changes in load relative to capacity (such as the ACWR) may indicate if the athlete is adequately prepared for the training load that is to be applied.^5,35^

While studies of associations between training load and injuries in adult team sports have increased in recent years,^18^ to date, only 2 studies have prospectively reported on injuries related to ACWR among adolescent competitive tennis players.^33,36^ However, these studies were relatively small and only investigated overall injury risk.

Our aims were to investigate if accumulated external workload spikes and high or low workload/age ratio were associated with the rate of shoulder complaints and shoulder injuries in competitive adolescent tennis players. Additional aims were to determine the incidence of injuries stratified for sex and level of play and to describe shoulder injury characteristics.

## Methods

### The SMASH Cohort Study

This study was based on data from the longitudinal cohort study called SMASH (Shoulder Management and Assessment Serving High Performance), performed in February 2018 to March 2019 in Sweden. Study participants (N = 301) were 13 to 19 years old, representing all 7 tennis regions in Sweden, recruited from the high-performance program supported by the Swedish Tennis Association. After informed consent, a baseline questionnaire was filled out before clinical testing. If <15 years of age, players’ legal guardian signed the consent form. Thereafter, players were followed with weekly questionnaires sent out each Sunday evening via an app with a reminder 24 hours later if a response had not yet been received, for 52 consecutive weeks.

### Baseline Measurements

The baseline questionnaire included questions about sex, age, tennis-related factors, history of shoulder problems (Oslo Sports Trauma Research Center Overuse Injury Questionnaire [OSTRC-O]),^8^ athletic identity, general health, sleep, and back/neck pain. The level of play was classified as regional and/or national level based on the Swedish Tennis Association high-performance program.

The study was performed in accordance with the Declaration of Helsinki and approved by the regional ethical review board (2012/1731/2 and 2018/2510).

### Study Population in This Study

**Figure 1.**
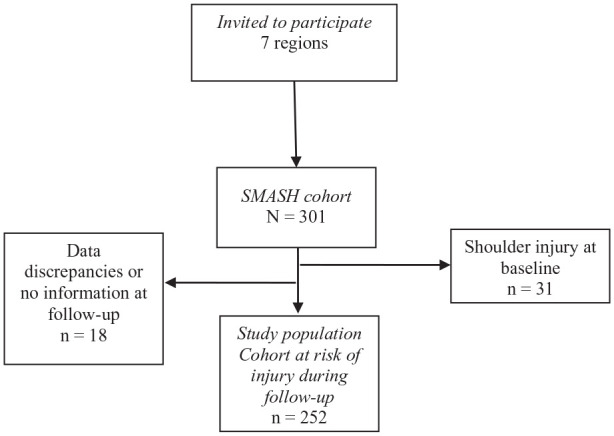
Flowchart describing the inclusion process.

For the risk analyses, only players without a shoulder injury (cutoff score 40/100 on OSTRC-O) in the 3 months preceding baseline testing, and that had answered any follow-up questionnaire, were included (n = 252). This was done to be able to study a population at risk of a shoulder injury. Figure 1 describes the inclusion process.

### Follow-up Measurements

Players were followed weekly regarding external workload (*How many hours and minutes match play have you performed the preceding week? How many hours and minutes have you practiced tennis on the tennis court the preceding week? How many hours and minutes have you performed activities that are not tennis related the preceding week?*), complaints/injuries in the shoulder (OSTRC-O), any acute injury, and number of training days per week. Information about the time-varying exposures as well as the time-varying outcome were collected from these weekly follow-up questionnaires.

*Exposure 1: Accumulated external workload spikes*: The weekly uncoupled ACWR using a rolling average were calculated by dividing the sum of training/match hours in the specific week with the mean number of training/match hours the preceding four weeks. Players with an ACWR >1.3 were classified as having an external workload “spike.”
*Exposure 2: Workload/age ratio*^24^: A workload/age variable was created with 3 levels: “reference category” : ratio = 0.90 – 1.10, “high” : ratio >1.10 (higher workload than age in years), and “low” : ratio <0.90 (lower workload than age in years). Workload in this variable was the mean of the total hours of tennis training/match play and fitness training in the preceding 4 weeks.
*Outcome:* The outcomes were a tennis-related shoulder complaint or injury measured with the OSTRC-O.^8^ A complaint was defined as sum score of at least 20/100 and an injury as a sum score of at least 40/100. Shoulder complaints based on OSTRC-O are a novel approach and were studied in addition to injuries to lower the risk of underreporting less severe but potentially important shoulder problems in this young population. In the risk analyses, only the incidence of a first complaint or injury were considered. For the estimation of the incidence of shoulder complaints/injuries over 52 weeks, recurrent events were considered. A player was classified as having a recurrent complaint/injury if he or she had at least 1 week without reporting an event, after having been classified as having a complaint or being injured.
*Confounders*: All risk analyses were adjusted for sex, age at baseline, playing level at baseline (national/regional), and number of days with training measured every week in the weekly follow-ups.

In the full SMASH cohort (N = 301), the average weekly response rate of the follow-up questionnaires was 85%, with 51% reporting complete data, 68% reporting at 90% of the follow-ups, 79% reporting 75% of the follow-ups, and 85% reporting at least 50% of the follow-ups.

### Statistical Analysis

When a weekly measurement was missing, last observation carried forward imputation was used. In the risk analysis, the imputed time points were omitted.

For the risk analyses, the frequency of “external workload spikes” (ACWR >1.3) was calculated, separately for tennis training/match, fitness training, and as a combined variable. Subsequently, the spikes were cumulated over time (from follow-up week 5). These factors along with the factor workload/age ratio were used in proportional hazards Cox regression models as time-varying covariates to determine the association with injury rates. Hazard rate ratios (HRR) with 95% CIs were analyzed by comparing exposed with unexposed players, where a player was considered to be at risk for an injury up until an injury occurred, until being censored or to the end of follow-up. The proportional hazards assumption held for all models (Schoenfeld residuals). In addition, a sensitivity analysis only of players with complete follow-up data (56% of the population) was conducted.

To investigate the importance of workload on propensity of being injured without considering the ACWR and spikes, the regression coefficients from a simple linear model for the past 4 weeks before the week in question (injury/not) were used to estimate the relationship of the downward/upward slope to the probability of being injured on week 5. As it was hypothesized that the relationship of the β-coefficient to injury is not linear, the coefficients were categorized, 1 of which was 0, and 4 others consisting of the positive and negative coefficients cut into 2 groups from their respective medians. The odds of injury were calculated with generalized estimation equation logistic regressions with exchangeable covariance structure.

Data management and analyses was done in R (Version 4.0.2; R Core Team, 2020; R Foundation for Statistical Computing) and Stata (Versions 15 and 16; StataCorp, 2017 and 2019; StataCorp LLC).

## Results

### Descriptive Analyses

Table 1 describes the baseline characteristics of the full SMASH cohort (N = 301) and stratified by shoulder injury status at baseline. The mean age was 14.4 years in players with no shoulder injury the preceding 3 months, and 57% of those were boys.

**Table 1.** Baseline characteristics by shoulder injury status (injury the preceding 3 months or not) at baseline

Baseline Characteristics	Shoulder Injury at Baseline (OSTRC-O score ≥40) (n = 31)	No Shoulder Injury at Baseline (n = 270)	*P*	All (N = 301)
Age, y, mean (SD)	15.4 (2.0)	14.4 (2.0)	0.01	14.5 (2.0)
Sex, male, % (n)	68 (21)	57 (155)	0.26	58 (176)
Height, cm, mean (SD)	173.5 (12.6)	169.3 (11.2)	0.05	169.8 (11.2)
Weight, kg, mean (SD)	62.7 (13.8)	57.8 (12.5)	0.04	58.3 (12.7)
BMI, kg/m^2^, mean (SD)	20.6 (2.8)	19.9 (2.5)	0.15	20.0 (2.5)
Passion for sport (AIMS), mean (SD)^*a*^	29.8 (3.3)	28.9 (3.7)	0.20	29.0 (3.7)
Quality of sleep, mean (SD)^*b*^	7.6 (2.0)	8.0 (1.7)	0.22	8.0 (1.7)
No. of hours of sleep per night, mean (SD)	7.7 (1.6)	8.2 (1.5)	0.08	8.1 (1.5)
General health, mean (SD)^*b*^	7.8 (1.7)	8.4 (1.7)	0.06	8.3 (1.7)
No. of matches in year 2017, mean (SD)	75.9 (44.7)	63.1 (33.7)	0.05	64.5 (35.1)
Hours per week of tennis training in year 2017, mean (SD)	10.6 (4.0)	9.4 (3.8)	0.10	9.5 (3.8)
Hours per week of fitness training in year 2017, mean (SD)	4.2 (2.4)	3.8 (2.5)	0.40	3.8 (2.5)
Normal racket tension, mean (SD)	23.8 (1.3)	23.4 (1.3)	0.11	23.4 (1.3)
One responsible tennis coach, yes, % (n)	58 (18)	68 (184)	0.26	67 (202)
One responsible fitness coach, yes, % (n)	45 (14)	64 (173)	0.04	62 (187)
Regularly performing rotation exercises for shoulder, yes, % (n)	65 (20)	56 (152)	0.34	57 (172)
Shoulder injury characteristics (n = 31), % (n)				
Injury onset: Gradual, mean (SD)	77 (23)	—		—
Injury onset: Acute, mean (SD)	23 (7)	—		—
Impairment on daily activities, mean (SD)	40 (12)	—		—
Impairment on sleep, mean (SD)	20 (6)	—		—
Stiffness in the shoulder, mean (SD)	73 (22)	—		—
Sought care for shoulder injury, mean (SD)	70 (21)	—		—

—, no data; AIMS, Athletic Identity Measurement Scale; BMI, body mass index; OSTRC-O, Oslo Sports Trauma Research Center Overuse Injury Questionnaire.

a Sum of total score of 7 items of the AIMS questionnaire (minimum 7, maximum 35) where high scores correspond to a high passion for sport.

b Rated on a numerical rating scale of 1 to 10 where 1 = very bad and 10 = very good.

Appendix Table A1 (available in the online version of this article) displays the baseline characteristics of the study population stratified by level of competition and sex, as well as the incidence of shoulder complaints/injuries per week and the number of cumulative external workload spikes over the 52-week follow-up. Regarding level of competition, the incidence of a shoulder complaint per week was 0.85 (95% CI, 0.55-1.31) in national players and 3.17 (95% CI, 2.82-3.55) in regional players. The corresponding incidences for a shoulder injury were 0.32 (95% CI, 0.16-0.65) and 1.33 (95% CI, 1.11-1.59), respectively. Regarding sex, the incidence of a shoulder complaint was 3.06 (95% CI, 2.68-3.50) in boys and 2.10 (95% CI, 1.73-2.56) in girls. The corresponding incidences for a shoulder injury were 1.20 (95% CI, 0.97-1.49) and 0.99 (95% CI, 0.74-1.32), respectively.

### Risk Analysis

An additional external workload spike was associated with an increased shoulder complaint and injury rate in all models (Table 2).

**Table 2.** The association between aspects of external workload and the incidence of shoulder injuries

	Shoulder Complaints (OSTRC-O Score ≥20)		Shoulder Injury (OSTRC-O Score ≥40)	
Training Profile	HRR	95% CI	HRR	95% CI
External workload spikes				
Accumulated external workload spikes in tennis training/match play, continuous variable	1.26^*a*^	1.13-1.40	1.26^*a*^	1.15-1.39
Accumulated external workload spikes in fitness training, continuous variable	1.11^*a*^	1.02-1.20	1.18^*a*^	1.09-1.27
Accumulated external workload spikes in fitness training and/or tennis training/match play, continuous variable	1.23^*a*^	1.12-1.36	1.22^*a*^	1.12-1.34
Workload/age ratio^*b*^				
0.9-1.1	1	—	1	—
<0.9	1.06^*a*^	0.40-2.82	0.66^*a*^	0.36-1.21
>1.1	1.64^*a*^	0.60-4.49	0.77^*a*^	0.39-1.54

—, no data; HRR, hazard rate ratio; OSTRC-O, Oslo Sports Trauma Research Center Overuse Injury Questionnaire.

a Adjusted for age, sex, level of competition, number of days with training/match per week in the preceding 4 weeks.

b The ratio between number of training hours in the preceding 4 weeks and age.

For each additional workload spike in tennis training/match play, the HRR was 1.26 (95% CI, 1.13-1.40) for a shoulder complaint and 1.26 (95% CI, 1.15-1.39) for a shoulder injury. For each additional workload spike in fitness training, the HRR was 1.11 (95% CI, 1.02-1.20) for a shoulder complaint and 1.18 (95% CI, 1.09-1.27) for a shoulder injury. Training workload/age ratio was not related to shoulder complaints or injuries.

To address the potential limitation of using the ACWR in the risk analyses,^23^ the association between a neutral β-coefficient and a negative and positive β-coefficient, respectively, from the linear model for the past 4 weeks of external workload before the week in question (injury/not) and a shoulder injury is presented in Appendix Table A2 (available online). The odds ratio of an injury in players with a positive slope was 7.57 (95% CI, 2.50-22.89).

### Incidence of Shoulder Complaints/Injuries in Tennis Training/Match Play

The incidence of shoulder complaints/injuries per 1000 hours of tennis training/match play in the risk cohort (n = 252) for all players and stratified by sex and level of play are presented in Table 3. In total, 90 players had a shoulder complaint and 44 players had a shoulder injury during the follow-up period in weeks 5 to 52. This corresponds to an incidence of a first shoulder complaint of 0.77 (95% CI, 0.67-0.85) and a first shoulder injury of 0.38 (95% CI, 0.30-0.48) for all players.

**Table 3.** Incidence and 95% CI of shoulder complaints/injuries per 1000 hours of tennis training/match play for all and stratified by sex and level of play

	All (n = 252)	National (n = 41)	Regional (n = 211)	Girls (n = 142)	Boys (n = 110)
Type of Incidence	n, Incidence (95% CI)	n, Incidence (95% CI)	n, Incidence (95% CI)	n, Incidence (95% CI)	n, Incidence (95% CI)
Shoulder complaints (cutoff ≥20) (including recurrent complaints) across 52 weeks per 1000 hours	312, 2.68 (2.40-2.99)	21, 0.85 (0.55-1.31)	291, 3.17 (2.82-3.55)	98, 2.10 (1.73-2.56)	214, 3.06 (2.68-3.50)
Shoulder injuries (cutoff ≥40) (including recurrent complaints) across 52 weeks per 1000 hours	130, 1.12 (0.94-1.32)	8, 0.32 (0.16-0.65)	122, 1.33 (1.11-1.59)	46, 0.99 (0.74-1.32)	84, 1.20 (0.97-1.49)
First shoulder complaint (cutoff ≥20) per 1000 hours	90, 0.77 (0.67-0.85)	11, 0.45 (0.29-0.69)	79, 0.87 (0.79-0.93)	36, 0.77 (0.66-0.90)	54, 0.77 (0.68-0.88)
First shoulder injury (cutoff ≥40) per 1000 hours	44, 0.38 (0.30-0.48)	5, 0.20 (0.09-0.44)	39, 0.42 (0.34-0.54)	16, 0.34 (0.23-0.51)	28, 0.40 (0.30-0.53)

Furthermore, in total, 312 shoulder complaints and 130 shoulder injuries, including recurrent injuries were reported during the follow-up period in the weeks 5 to 52. This corresponds to an incidence per 1000 hours of tennis training/match play of a shoulder complaint of 2.68 (95% CI, 2.40-2.99) and a shoulder injury of 1.12 (95% CI, 0.94-1.32) for all players.

To address the potential limitations of using accumulated spikes in the risk analyses, the incidence of at least 1 shoulder complaint/shoulder injury across the 52 weeks, stratified for numbers of spikes, is presented in Appendix Table A3 (available online). The incidence of an injury was 0.010 (95% CI, 0.008-0.012) per week.

## Discussion

Our main findings indicate that external workload spikes in tennis training, tennis match play, or fitness training are associated with a higher incidence of shoulder complaints/injuries in competitive adolescent tennis players. We did not find any associations between the workload/age ratio and the incidence of shoulder complaint/injury, indicating that in terms of injury risk, adaptation of workload to age may not be important in this age span. Furthermore, the results revealed a higher incidence of overall shoulder complaints/injuries in boys and in regional players. There are some differences in baseline characteristics between injured and uninjured players, such as height, weight, sleeping patterns, and coaching, that may constitute potential risk factors to address in future studies.

Previous studies, mainly of team sports, showed that the ACWR was associated with the risk of injury.^4,21,27^ To the best of our knowledge, the only previous studies to analyze the ACWR in tennis, included (1) high-performance junior players (n = 15) and (2) intermediate to advanced junior players (n = 26).^33,36^ The study of high-performance junior players presented a risk ratio of 2.29 (1.03-5.07) of any injury,^33^ and the other study investigating ACWR in intermediate to advanced players presented an overall injury HRR of 2.76 (1.58-4.82).^36^ Although comparisons with our results are difficult since training load, sample size, follow-up time, and classification of injury differ, we judge that our main results are similar with previous findings.

The boys in our study reported a higher overall incidence of shoulder complaint/injury than the girls. The results are in line with a study of elite tennis players^32^ but in contrast to another study of elite junior tennis players.^37^ Additionally, the boys reported a higher training load (13.1 h/wk) than the girls (11.5 h/wk), which may contribute to the higher incidence.

National players reported a lower incidence of overall shoulder complaints and injury as well as lower incidence for first injury. In this regard, regional players showed a ~4 times higher incidence of shoulder complaint/injury than their peers at the national level.

With regard to spikes in training load, fewer national players (12%) than regional players (28%) reported cumulative spikes in the range >10, meaning that when building chronic load, national players had more consistent training load from week to week. From a clinical perspective, players among the national squad had more frequent access to individual tennis coaching, specialized strength and conditioning coaches, sports medicine staff, and an overall more professional environment, which may contribute to the lower incidence of shoulder complaints and injury despite a higher training volume (+3.3 h/wk and 4.3 h/wk) for boys and girls, respectively.

In addition to workload and injuries, an independent risk of overuse injury has been reported in (1) young athletes who specialize early in a single sport and (2) those who spend numerically more hours per week participating in sports versus their age in years.^24^ However, in our study, no such association between workload and age was evident.

### Methodological Discussion

In adolescent athletes, it is crucial not to overlook early detection of injuries.^10^ In that regard, the OSTRC-O system of reporting overuse injuries, with a cutoff score of 40/100, has become a frequently used method.^7,8^ However, this threshold may need some consideration in adolescent athletes given that injury as burnout is common in sports involving early specialization.^26^ First, adolescent athletes have less experience in understanding pain and may believe pain is normal, or they may believe that acknowledging pain might stop them from participating in sport, resulting in their underreporting pain.^10^ Moreover, from a loading perspective, their tendonous mechanical properties do not develop as fast as their muscle strength, therefore an inherent risk of developing more severe injuries over time is evident in the adolescent athlete.^30^ Therefore, to minimize the risk of underreporting complaints/injuries in a younger athletic population, and to protect the health and well-being of these athletes, we analyzed 2 different cutoff scores of the OSTRC-O, ≥20 and ≥40, to investigate the potential different outcome of a lower threshold in adolescent athletes.

There has been some critique of the methods used for estimating the risk of injury related to the ACWR, as there has been a lack of studies with a prospective design to determine the causal effect of ACWR on injuries.^42^ One of the strengths of the current study is the large size and the longitudinal design. In addition, the methodological problem associated with investigating recurrent injuries was addressed by focusing solely on index complaints and injuries.^42^ Furthermore, only players without a shoulder injury were included—only the first complaint/injury was considered to avoid the risk of reversed causality, even though injured players also may have a higher risk of recurrent injuries if exposed to ACWR spikes.

Wang et al^42^ suggested that the uncoupled ACWR, as was used in this study, should be preferred over the coupled ACWR, but that this method may still not be optimal to measure changes in workload, as it may obscure weekly variations in training load. Such nondifferential misclassification may dilute the true association between the exposure and outcome. In contrast, the use of the valid and reliable OSTRC-O lowered the risk of a misclassification.^8,14^

In the categorization of a workload “spike,” the threshold >1.3 was chosen based on our clinical experience with regard to adolescents that most likely do not tolerate as large changes in workload as adult athletes. However, it is possible that another cutoff may have yielded a slightly different result.

Another limitation is the potential underestimation of the association between workload spikes and injury incidence that may arise from the cumulated external workload spikes over time and the use of the accumulation as a time-varying covariate in the analyses. As the spikes can only cumulate with time, the estimates cannot be taken as correct estimates of the effect, and the actual effect may be larger than estimated. To illustrate this potential underestimation, we estimated the incidence of injuries at the different “stages” of spike accumulation in relation to the total follow-up time in that state (Appendix Table A3, available online). The decrease in incidence with the higher the cumulative number of spikes indicates an underestimation of the associations.

There is also an issue of unmeasured time-varying confounding when investigating time-varying exposures in causal models.^42^ In the present study, the only time-varying potential confounder was the number of days with training per week. Even though the analyses were also adjusted for several other potential confounders from baseline, unmeasured confounding from sleep quality, health, stress, and other risk factors for injury may be present.

To further investigate the effect of training/playing pattern on propensity of being injured, without using the ACWR, and to avoid the use of a ratio in risk analyses,^23^ we estimated the relationship of the regression coefficients from a simple linear model for the past 4 weeks before the week in question (injury or not) to the probability of being injured on week 5. These analyses indicate that a stability of the workload during the last 4 weeks was protective for injury in week 5 (Table 2). However, the confidence intervals were very wide, partly because of the small number of injuries in 4 weeks.

A strength of this study is the average weekly response rate of 85%. A sensitivity analysis conducted on players who had follow-up data for all 52 weeks (56% of the population at risk) produced similar results to the main analysis. This, in combination with the fact that our study population included the vast majority of the adolescent tennis players at the regional and national level in Sweden, indicate a low risk of selection bias and that the external validity is high. The characteristics in the full SMASH cohort was very similar to the characteristics in the risk cohort we have investigated regarding the incidence of injuries (Table 1). Nevertheless, despite the relatively large sample size, we have limited statistical power for the risk analyses. Last, as a comparison with the analyses of ACWR spikes as a potential risk factor, the 5-week average hours of training, categorized as low or high based on the median value, was modeled. The HRR of an injury in players with high chronic load was not higher than in players with low chronic load (not shown in table).

In summary, after having considered strengths and limitations, we believe that the associations found in this study are valid, even though the causal chains are complex, precluding strong statements about causality.

## Conclusion

Accumulated external workload spikes of tennis training, match play, and/or fitness training are associated with a higher rate of shoulder complaints and shoulder injuries in competitive adolescent tennis players. Workload/age ratio was not associated with the incidence of shoulder complaint or injury in this study.

### Practical Implications

To reach the professional level of tennis (ATP/WTA), adolescent tennis players need to submit to high volumes of tennis and fitness on a weekly basis over a long period of time. Maintaining continuity in daily training provides young tennis players with the opportunity to develop their physical and technical abilities, thereby facilitating robustness and resilience. Nevertheless, such specialized training can come with the threat of overuse injury.^24^ Since there are still some uncertainties regarding load management in adolescent sports to prevent injuries,^9^ it is an area in need of more research. However, based on our finding of greater risk of shoulder complaints and injury with spikes in workload, we suggest that a well-planned, periodized training program that safely builds chronic load may be an important and proactive intervention. Finally, successful management of overuse injury includes early diagnosis, intervention, and rehabilitation. Based on our investigation, we suggest that practitioners working with adolescent tennis players should incorporate a lower OSTRC-O cutoff score of >20 to capture minor shoulder complaints before they develop into more severe shoulder injuries.

## Supplemental Material

sj-docx-1-sph-10.1177_19417381211051643 – Supplemental material for Association Between Spikes in External Training Load and Shoulder Injuries in Competitive Adolescent Tennis Players: The SMASH Cohort Study Click here for additional data file. Supplemental material, sj-docx-1-sph-10.1177_19417381211051643 for Association Between Spikes in External Training Load and Shoulder Injuries in Competitive Adolescent Tennis Players: The SMASH Cohort Study by Fredrik Johansson, Ann Cools, Tim Gabbett, Jaime Fernandez-Fernandez and Eva Skillgate in Sports Health: A Multidisciplinary Approach
