# Supplementary material for: Association Between Spikes in External Training Load and Shoulder Injuries in Competitive Adolescent Tennis Players: The SMASH Cohort Study
Source: Sports Health. 2021 Oct 25;14(1):103–10. doi: 10.1177/19417381211051643 (PMC8655479; doi:10.1177/19417381211051643)
Supplement: sj-docx-1-sph-10.1177_19417381211051643 – Supplemental material for Association Between Spikes in External Training Load and Shoulder Injuries in Competitive Adolescent Tennis Players: The SMASH Cohort Study [file sj-docx-1-sph-10.1177_19417381211051643.docx]

**APPENDIX**

**Table A1**. Characteristics and incidence of shoulder injuries and complaints by sex and level of competition (n=252)

| Baseline characteristics | National | Regional | Boys | | | Girls | | |
| --- | --- | --- | --- | --- | --- | --- | --- | --- |
|  | **All**  (n=41) | **All**  (n=211) | **All**  (n = 142) | **National**  (n =18) | **Regional**  (n = 124) | **All**  (n = 110) | **National**  (n = 23) | **Regional**  (n = 87) |
|  |  |  | **Mean (SD)** | **Mean (SD)** | **Mean (SD)** | **Mean (SD)** | **Mean (SD)** | **Mean (SD)** |
| Age, years^a^ | 13.9 (1.5) | 14.4 (2.0) | 14.4 (2.0) | 14.1 (1.6) | 14.5 (2.0) | 14.2 (1.9) | 13.7 (1.54) | 14.4 (2.0) |
| Height, cm ^a^ | 168.2 (10.7) | 168.8 (10.0) | 171.7 (12.1) | 174.2 (9.9) | 171.3 (12.3) | 165.0 (7.9) | 163.7 (9.1) | 165.4 (7.6) |
| Weight, kg^a^ | 58.4 (11.3) | 57.0 (12.4) | 59.3 (13.6) | 61.2 (12.4) | 59.0 (13.8) | 54.7 (9.7) | 56.3 (10.2) | 54.3 (9.6) |
| BMI, kg/m^a^ | 20.5 (2.7) | 19.7 (2.4) | 19.8 (2.4) | 20.0 (2.5) | 19.8 (2.4) | 19.9 (2.6) | 20.9 (2.8) | 19.7 (2.5) |
| Hours per week of tennis training/match play^b^ | 12.2 (6.0) | 9.3 (5.5) | 10.5 (5.4) | 12.9 (4.6) | 10.1 (5.4) | 8.8 (5.9) | 11.6 (6.9) | 8.1 (5.3) |
| Hours per week of fitness training^b^ | 3.2 (2.0) | 2.4 (1.9) | 2.7 (12.1) | 3.0 (2.0) | 2.6 (1.8) | 2.6 (1.8) | 3.2 (2.1) | 2.5 (2.1) |
| Hours per week of training, all^b^ | 15.3 (6.5) | 11.8 (6.1) | 13.1 (6.0) | 16.0 (5.1) | 12.7 (6.0) | 11.5 (6.6) | 14.9 (7.5) | 10.6 (6.0) |
| Mean number of days with training per week^c^ | 5.5 (1.7) | 5.1 (1.8) | 5.3 (1.7) | 5.6 (1.7) | 5.3 (1.7) | 5.0 (1.9) | 5.4 (1.7) | 4.9 (1.9) |
| Number of years playing tennis | 8.5 (2.0) | 8.4 (2.7) | 8.6 (2.5) | 8.9 (1.4) | 8.6 (2.7) | 8.2 (2.6) | 8.3 (2.3) | 8.2 (2.7) |
| Shoulder injury per week over 52 weeks, cut-off >20, n  Incidence (95% CI) | 21  0.85  (0.55 – 1.31) | 291  3.17  (2.82 – 3.55) | 214  3.06  (2.68 – 3.50) | 7  0.61  (0.29 – 1.28) | 207  3.54  (3.09 – 4.05) | 98  2.10  (1.73 – 2.56) | 14  1.06  (0.62 – 1.78) | 84  2.52  (2.03 – 3.12) |
| Shoulder injury per week over 52 weeks, cut-off **≥**40; n, Incidence, (95% CI) | 8  0.32  (0.16 – 0.65) | 122  1.33  (1.11 - 1.59) | 84  1.20  (0.97 – 1.49) | 1  0.09  (0.01 – 0.62) | 83  1.42  (1.14 – 1.76) | 46  0.99  (0.74 – 1.32) | 7  0.53  (0.25 – 1.11) | 39  1.17  (0.85 – 1.60) |
| Total number of cumulative spikes in fitness training and/or tennis training/match play during 52 weeks, % (n)  0  1-10  >10 | 15% (6)  73% (30)  12% (5) | 15% (32)  57% (118)  28% (58) | 19% (26)  59% (83)  22% (31) | 28% (5)  72% (13)  - (-) | 17% (21)  57% (70)  26% (31) | 11% (12)  60% (65)  29% (32) | 4% (1)  74% (17)  22% (5) | 13% (11)  56% (48)  31% (27) |

^a^From baseline questionnaire/clinical screening

^b^From weekly follow-up: Mean over 52 weeks, of the mean number the preceding four weeks

^c^From weekly follow-up: Mean of the mean number of days of tennis/fitness training hours, match-play hours or any other sport the preceding four week

**Table A2.** The odds ratio (OR) between a neutral β-coefficient and a negative and positive β-coefficient respectively for a shoulder injury.

|  | **Shoulder injury**  **(OSTRC ≥ 40)** | |
| --- | --- | --- |
| Workload^b^ | **OR**^a^ | **(95% CI)** |
| Neutral β-coefficient (no slope) | 1 | **-** |
| Negative β-coefficient (slope down) | 3.77 | 1.11 – 12.79 |
| Slightly negative β-coefficient (slope slightly down) | 5.83 | 1.84 - 18.54 |
| Slightly positive β-coefficient (slope slightly up) | 3.30 | 0.94 - 11.61 |
| Positive β-coefficient (slope up) | 7.57 | 2.50 - 22.89 |

^a^Odds Ratio (OR) calculated by with generalized estimation equations (GEE) logistic regression with exchangeable covariance stricture adjusted for age, sex, level of competition

^b^The regression coefficients from a linear model for the last four weeks external workload (tennis training, match play and fitness) before the week in question (injury/not) and the estimation of the relationship of the downwards/upwards slope to the probability of being injured on week 5.

**Table A3.** The incidence with 95% Confidence Intervals (95% CI) of at least one shoulder complaint or shoulder injury across 52 weeks, stratified for numbers of spikes, sex and playing level.

| Cumulative numbers of spikes | **Person time in weeks** | | **Number of cases of shoulder complaints/injury** | | **Incidence of shoulder complaints/injury**  **(95% CI)** | |
| --- | --- | --- | --- | --- | --- | --- |
|  | **Shoulder complaints**  **(OSTRC ≥20)** | **Shoulder injury**  **(OSTRC ≥40)** | **Shoulder complaints**  **(OSTRC ≥20)** | **Shoulder injury (OSTRC≥40)** | **Shoulder complaints (OSTRC ≥20)** | **Shoulder injury**  **(OSTRC ≥ 40)** |
| 0-3 | 3119 | 3372 | 35 | 8 | 0.011  (0.08 – 0.016) | 0.002  (0.001 – 0.005) |
| 4-5 | 3836 | 4702 | 37 | 20 | 0.010  (0.07 – 0.013) | 0.004  (0.001 – 0.07) |
| 6-9 | 1769 | 2340 | 14 | 13 | 0.008  (0.05 – 0.013) | 0.001  (0.003 – 0.009) |
| 10-15 | 547 | 733 | 4 | 3 | 0.007  (0.03 – 0.019) | 0.006  (0.003 – 0.030) |
| 15-19 | 75 | 108 | 0 | 0 | 0 (-) | 0.004  (0.001 – 0.013) |
| ≥20 | 5 | 5 | 0 | 0 | 0 (-) | 0 (.) |
| Total, all | 9531 | 11260 | 90 | 44 | 0.010  (0.008 – 0.012) | 0.004  (0.003 – 0.005) |
| Total, girls | 4270 | 5126 | 36 | 16 | 0.008  (0.006 – 0.012) | 0.003  (0.002 – 0.005) |
| Total, boys | 5081 | 6134 | 54 | 28 | 0.011  (0.008 – 0 014) | 0.005  (0.003 – 0.007) |
| Total, national players | 1812 | 2033 | 11 | 5 | 0.006 (0.003 – 0.011) | 0.002  (0.001 – 0.006) |
| Total, regional players | 7539 | 9227 | 79 | 39 | 0.010  (0.008 – 0.013) | 0.004  (0.003 – 0.006) |
